# Chemical Denervation to Relieve Symptoms in Jugular Venous Compression Syndrome: A Case Report

**DOI:** 10.1155/crvm/9186091

**Published:** 2025-02-17

**Authors:** Wesley Paulson, Morgan A. Voulo, Shivani Patel, Andrew J. Rothka, Sarahrose Jonik, Neyha Cherin

**Affiliations:** ^1^Department of Physical Medicine and Rehabilitation, Penn State College of Medicine, Hershey, Pennsylvania, USA; ^2^Department of Physical Medicine and Rehabilitation, Penn State Hershey Rehabilitation Hospital, Hummelstown, Pennsylvania, USA

**Keywords:** blood circulation, botulinum toxin, case report, jugular veins, quality of life

## Abstract

Jugular venous compression syndrome (JVCS) is caused by internal jugular vein compression, leading to headaches, neck discomfort, tinnitus, vertigo, confusion, and blurred vision. These impairments can diminish functional outcomes and compromise quality of life for patients. Literature-based treatments focus on surgical approaches and do not include chemodenervation. However, chemodenervation may be an additional treatment modality to consider. We present the first published case of chemodenervation utilized to successfully treat debilitating neck pain, headaches, and vertigo symptoms for JVCS, resulting in improved functionality and quality of life. This chemical denervation to the anterior neck musculature was trialed in hopes of avoiding surgical intervention. After three treatments, significant symptomatic relief with improved ability to work, improved performance of activities of daily living, and enhanced quality of life were noted. Educating physicians about JVCS and the various treatment modalities available is essential, as less invasive treatment options may not only become available to assist with the functional component of the condition but could also serve as potential alternatives to surgical and endovascular management in carefully selected patients, with the goal of optimizing function and improving quality of life among patients.

## 1. Introduction

Jugular venous compression syndrome (JVCS) is a rare condition that occurs when the jugular vein is compressed from various etiologies, obstructing normal blood flow away from the brain [1]. Compression can produce a variety of symptoms ranging from headache, neck discomfort, and tinnitus, to debilitating vertigo, confusion, and blurred vision [2–5]. Among reported cases in the literature, patients note that this syndrome has significant negative effects across multiple domains including job performance, ability to sleep, and overall quality of life [6, 7]. Specific head and neck positioning can trigger varying degrees of compression of the jugular vein [1]. Given the extensive length of the jugular vein, multiple sites for compromise are possible. For example, structures in the neck, such as the styloid process, scalene musculature, omohyoid muscle, and the internal carotid artery, have all been identified as potential sources of jugular vein compression [1–3]. A review of the literature reveals that the primary treatments for JVCS are generally surgical and endovascular and based on case-specific anatomical involvement [1, 5]. Multiple systematic reviews found that surgical and endovascular treatment provided the greatest success [4, 5]. Though there is an existing body of evidence regarding the efficacy of surgical and endovascular interventions for JVCS, patients may prefer a more conservative approach [4, 5]. After a thorough literature review, evidence is lacking regarding the utility of chemodenervation to treat symptoms associated with JVCS. The authors report a case of a 61-year-old female who presented to our Physical Medicine and Rehabilitation (PM&R) clinic after 5 years of ongoing and debilitating symptoms related to JVCS to explore nonsurgical options for symptom control.

## 2. Case Presentation

The patient's journey began in 2008 following a sudden blow to her head and neck while body surfing. Weeks after the accident, she developed hoarseness and vocal cord weakness. At that time, she was diagnosed with Eagle syndrome (ES), a disorder involving compression of anterior neck structures from an abnormally long or aberrantly angled styloid process [1, 8]. It was believed that the body surfing injury triggered abnormal growth of her right styloid process impacting neighboring anatomy with neck movement. She underwent a right styloidectomy resulting in the return of her voice.

In 2019, after a soft tissue neck massage, the patient experienced sudden-onset right-sided hearing loss, tinnitus, and increased pressure, along with debilitating vertigo and headaches. Bilateral neck rotation and supine positioning exacerbated her symptoms, making activities of daily living (ADLs) difficult. Medical evaluation included a negative CT of the head, a negative Epley maneuver, an audiogram in October 2019 revealing right sensorineural hearing loss, and three transtympanic steroid injections. Additionally, vestibular function testing included electronystagmography, vestibular evoked myogenic potential assessment, and a video head impulse test assessment, which were all normal. She received a bone anchored hearing aid in the fall of 2019 and ultimately received a cochlear implant on the right side.

In March of 2020, a CT angiogram of the neck revealed focal flattening of the mid left vertebral artery at the C4–C5 level resulting in approximately 75% stenosis at this location, which was read as being consistent with the patient's history of rotational vertebral artery occlusion syndrome by radiology. Consequently, this led to the diagnosis of bowhunter's syndrome (BHS), a condition resulting in vertebrobasilar insufficiency or vertebral artery ischemia from neck movements such as rotation from left to right [1]. Given the known risk of severe neurologic compromise, including stroke, a C5-C6 left vertebral artery decompression and anterior cervical discectomy and fusion were performed. Eight weeks postop, the patient's cervical collar was removed, and she reported resolution of vertigo with leftward head rotation but continued to endorse vertigo with rightward head rotation while lying in the supine position.

**Figure 1:**
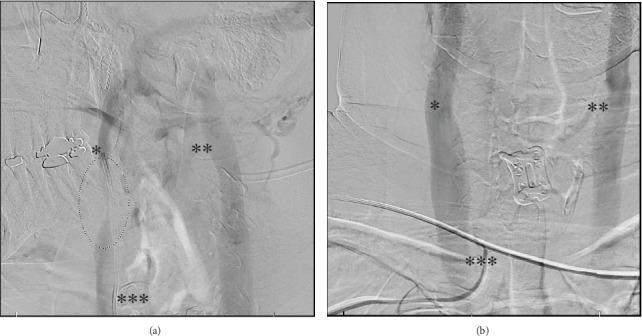
Cerebral angiogram with right vertebral artery injections. (a) Digital subtraction angiography (DSA) with 90° head rotation to the right. Right internal jugular vein is marked with ∗, and left internal jugular vein is marked with ∗∗. Catheter in the right vertebral artery is marked with ∗∗∗. Dashed circle highlights the area of compression. (b) DSA with head in neutral position. The right internal jugular vein is marked with ∗, and left internal jugular vein is marked with ∗∗. Catheter in the right vertebral artery is marked with ∗∗∗.

Due to ongoing debilitating vertigo and headaches, the patient sought additional medical advice and ultimately underwent three neck venograms in 2022. Two of the venograms were positive for rotational compression. The first was a cerebral angiogram, which showed “dynamic stenosis of the right internal jugular vein at the skull base with the patient's head rotated to the right, resolving with the head in a neutral position” (see Figure 1). The second was from a right internal jugular vein injection, neck, and 3D views: “redemonstration of moderate to severe stenosis of right internal jugular vein at the skull base with provocative maneuvers.” The third, a cerebral angiogram/venogram, showed “no significant narrowing of the right internal jugular at the level of the skull base and high cervical spine.” The patient had described her vertigo as also being impacted by arm positioning in addition to head position and had said her arm position was different on the third study, compared to the second venogram.

Further evaluation in 2023 included a CT venogram of the head and neck with contrast, with variation in head position, which showed only mild narrowing of both internal jugular veins, with no difference based on head positioning. A quantitative ultrasound of the arterial and venous system in the central veins of the upper extremities using photoplethysmography revealed a dynamic left subclavian vein compression depending on left arm positioning.

At that point, it was unclear if there was dynamic compression of the right internal jugular vein. The history provided by the patient strongly suggested it, while the imaging studies provided a more mixed picture, with two of the three initial venograms demonstrating dynamic compression, while the venogram in 2023 did not suggest dynamic compression of the right internal jugular vein. Thus, a diagnostic lidocaine block to the right sternocleidomastoid muscle (SCM) and anterior scalene musculature was trialed to see if this helped her symptoms. It resulted in improvement of her vertigo and of her headaches and was followed by a repeat injection of the SCM alone which did not improve her vertigo or headache. Given the transient relief experienced as a result of the nerve block, the patient was ultimately referred to our PM&R clinic for “botulinum toxin injections to the neck musculature to improve pain” from underlying JVCS. She was also prescribed physical therapy at least once per week. Physical therapy consists of 15-min Graston therapy, followed by stretching of the right trapezius, SCM, and the scalene musculature, with dry needling of the trapezius, SCM, and the right three scalene muscles.

When the patient presented for initial evaluation to our clinic in 2023, she endorsed 5 years of the following impairments: vertigo, tinnitus, headache, blurred vision, neck discomfort, sleep disturbance, impaired balance, and progressively worsening functionality. At that point, the patient had trialed gabapentin, duloxetine, and 6 months of vestibular rehabilitation. Duloxetine helped reduce the intensity of her chronic headaches, but none of the interventions alleviated or improved the vertigo.

Our team decided to proceed with a stepwise approach regarding onabotulinumtoxinA injections for her JVCS. She has received four injections to date. With onabotulinumtoxinA, in sequential order and at 3-month intervals, the injections were as follows: (1) 20 units to the right anterior scalene, (2) 20 units to the right anterior scalene and 15 units to the right SCM, (3) 20 units to the right anterior scalene and 20 units to the right SCM, and (4) 25 units to the right anterior scalene and 20 units to the right SCM.

There was no symptomatic relief after the first injection. She began to see relief of her vertigo and headaches after the second injection. Since that time, she has had significant relief of vertigo with head rotation to the right and in the supine position and a significant decrease in frequency and intensity of headaches. Headache relief starts to take effect 1 week after each subsequent injection. Since beginning this treatment regimen, she has experienced significant gains in her ability to function both at work and at home, thereby improving her quality of life. Her headaches start to return about 10 weeks after the onabotulinumtoxinA injection.

In addition to the onabotulinumtoxinA injections every 3 months, she continues physical therapy appointments every other week until the onabotulinumtoxinA starts to wear off, then increases the physical therapy appointments to weekly. For her headaches, she takes 20 mg duloxetine daily, as needed ibuprofen 600 mg, and as needed sumatriptan 50 mg.

## 3. Discussion

JVCS is defined as compression of the jugular vein by various structures in the surrounding neck anatomy [1]. Due to the rarity of JVCS and the various anatomical etiologies of the condition, there is significant heterogeneity in the terminology that is used to define this syndrome, resulting in confusion in diagnosis and treatment approach [1–3]. Such terms include dynamic jugular vein stenosis, jugular bow hunter's syndrome, jugular compression syndrome, and jugular venous outflow disturbance [1, 2, 7]. In line with the incongruity of how this phenomenon is described, the current literature regarding treatment is sparse and largely consists of systematic reviews of surgical and interventional case reports [1, 3, 5]. However, this is a growing area of research, with many of the existing articles calling for additional research into internal jugular vein stenosis in terms of clinical manifestations, diagnosis, and nonsurgical treatment modalities [7, 9]. Given the promising results of symptom relief following chemodenervation seen in our patient, further research efforts could further explore alternatives to the typical surgical and endovascular approaches, for treating the debilitating symptoms associated with JVCS. With more research, this could potentially benefit carefully selected patients who either have contraindications to surgery and endovascular interventions or who wish to pursue a less invasive option first.

As described, the vast majority of currently published treatments for cases of JVCS involve either surgery and/or angioplasty [4, 5]. However, there is benefit in exploring conservative treatment modalities as alternative options may exist for patients with this condition. Having a nonsurgical and noninterventional approach not only benefits patients who may not be surgical or interventional candidates but also benefits those who may prefer more conservative management. While surgical interventions are not without risk such as infection, bleeding, vascular injury, and nerve injury, one must also consider the financial impact and time for recovery patients endure [4]. In the literature we examined, while the surgical interventions appeared to carry more risk, the endovascular risk of stenting for JVCS includes possible migration of the stent, thrombosis, potential need for lifelong anticoagulation, and nerve injury [4, 5]. Unsurprisingly, chemical denervation is not without risk as well. Some of these risks include infection, bleeding, and possible distal spread of the toxin, and all must be considered prior to pursuing this treatment route. Chemical denervation requires this patient to return to clinic every 3 months for a repeat injection. When weighing the risks and benefits between surgical, endovascular, and nonsurgical options, chemical denervation could serve as a more favorable alternative for some patients. Thus, more research should be done to determine if chemical denervation could be part of the treatment options for well-selected patients in this population.

In this patient's case, chemical denervation to the right-sided anterior neck muscles proved to be a successful approach to reduce symptoms without the patient requiring another surgery. OnabotulinumtoxinA is currently approved for many noncosmetic cervical manifestations including dystonia, temporomandibular joint (TMJ) disorders, sialorrhea, masticatory myalgia, and migraine headache [10]. To the authors' knowledge, this case is the first to report the utility of onabotulinumtoxinA in managing symptoms experienced by a patient with JVCS.

The case presented shows symptomatic benefit in a single patient. There are several unknowns in this case report that should be noted. Muscular tension in the SCM can lead to vertigo irrespective of venous compression [11]. The patient has not had a repeat venogram since the onabotulinumtoxinA injections; thus, it is not absolutely certain that her symptomatic improvement is due to decreased venous compression. Secondly, due to the limited literature and experience with onabotulinumtoxinA injections in patients with JVCS, there is no long-term data regarding this treatment modality. Therefore, until additional empiric data are obtained, the approach in this case report cannot be applied as a general recommendation.

These limitations should not take away from the promising results seen in this patient who suffered for years with debilitating symptoms. Future studies investigating the efficacy of chemodenervation with onabotulinumtoxinA injections for well-selected patients with JVCS are needed. Comparing the outcomes of patients undergoing injections versus the outcomes of surgical and interventional management is warranted. The patient in this case had a very thorough evaluation with a variety of experts in vascular medicine, venous compression, and the treatment of vertigo, and her diagnostic workup included specifically establishing muscular involvement in her symptomatology before this management strategy was implemented. This case encourages further research into the use of chemical denervation in carefully selected patients with JVCS.

## 4. Conclusion

JVCS is a condition unfamiliar to many physicians. However, it leads to significant limitations in physical, psychosocial, and functional domains among patients. This report describes a single case of chemical denervation applied to treat debilitating symptoms of JVCS in a patient who had undergone a thorough workup for the condition. Significant relief was obtained after three rounds of chemical denervation, and the patient reported improvement in her ability to participate in work, ADLs, and quality of life. Published reports of patients with JVCS are limited to surgical and endovascular interventions as the primary treatment of choice and do not include chemodenervation. Multimodal treatment approaches are key in healthcare, and one treatment option does not always apply to all. Exploring alternative nonsurgical and noninterventional modalities for JVCS, particularly for patients with advanced age and multiple comorbidities and those who are uninterested in surgical interventions and who may not be candidates for endovascular intervention, would be helpful. Chemical denervation is not currently FDA approved for JVCS. Further research would be needed to determine if it is a suitable and effective nonsurgical approach for helping improve patients' symptoms, level of functioning, and ultimately quality of life.

## Data Availability

Data sharing is not applicable to this article as no new data were created or analyzed in this study.
